# Vaccinia virus H7-protein is required for the organization of the viral scaffold protein into hexamers

**DOI:** 10.1038/s41598-022-16999-2

**Published:** 2022-07-29

**Authors:** Susanne Tonnemacher, Marcia Folly-Klan, Anastasia D. Gazi, Simon Schäfer, Esthel Pénard, Regina Eberle, Renate Kunz, Paul Walther, Jacomine Krijnse Locker

**Affiliations:** 1 Electron Microscopy of Pathogens, Paul Ehrlich Institute, Paul Ehrlichstreet 51-59, 63225 Langen, Germany; 2 Ultrastructural Bio-Imaging Unit, Institut Pasteur, 28, rue du Dr. Roux, 75015 Paris, France; 3 Central Facility for Electron Microscopy, Ulm University, 80981 Ulm, Germany; 4 Justus Liebig University, Giessen, Germany

**Keywords:** Cell biology, Microbiology, Cellular microbiology, Pathogens, Virology, Structural biology, Electron microscopy

## Abstract

Viruses of the giant virus family are characterized by a structurally conserved scaffold-capsid protein that shapes the icosahedral virion. The vaccinia virus (VACV) scaffold protein D13, however, transiently shapes the newly assembled viral membrane in to a sphere and is absent from the mature brick-shaped virion. In infected cells D13, a 62 kDa polypeptide, forms trimers that arrange in hexamers and a honey-comb like lattice. Membrane association of the D13-lattice may be mediated by A17, an abundant 21 kDa viral membrane protein. Whether membrane binding mediates the formation of the honey-comb lattice or if other factors are involved, remains elusive. Here we show that H7, a 17 kDa protein conserved among poxviruses, mediates proper formation of D13-hexamers, and hence the honey comb lattice and spherical immature virus. Without H7 synthesis D13 trimers assemble into a large 3D network rather than the typical well organized scaffold layer observed in wild-type infection, composed of short D13 tubes of discrete length that are tightly associated with the endoplasmic reticulum (ER). The data show an unexpected role for H7 in D13 organization and imply that formation of the honey-comb, hexagonal, lattice is essential for VACV membrane assembly and production of infectious progeny. The data are discussed with respect to scaffold proteins of other giant viruses.

## Introduction

Enveloped viruses acquire their membrane from the host, via budding at cellular membranes or by wrapping, acquiring a single or double membrane(s), respectively^1^. However, for the large DNA-virus vaccinia virus (VACV), member of the poxviridae, the origin and biogenesis of its membrane remains controversial until this date.

By electron microscopy (EM) newly synthesized VACV membranes first appear as short, half-moon shaped units, the crescents, that grow into membrane spheres, the immature virus (IV). Upon DNA-uptake the IV matures in to the brick-shaped mature virus (MV) that is infectious^2^. The formation of the crescent membrane and spherical IVs critically depends on the viral scaffold protein, the gene product of D13, a 62 kDa protein. It assembles on the convex side of the growing viral membrane mediating curvature and in the absence of D13 crescents, IVs and consequently MVs, fail to form^3^. In infected cells D13 forms a regular structure on the surface of the viral membrane. It is composed of hexagons of D13 trimers, arranged in a high-order lattice resembling a honey comb^4,4^. Based on electron tomography (ET) we proposed that small patches of the honey-comb lattice preassemble in the cytoplasm and then recruit vesicles of roughly 50 nm in diameter containing viral membrane proteins^6^. The vesicles are opened by membrane rupture and contribute to the formation of IV-membrane, an open lipid bilayer shaped by D13 on its convex side. Association of D13 with viral membranes may be mediated by the gene product of A17, a major viral membrane protein of 21 kDa^7,8^; it is synthesized in the endoplasmic reticulum and locates to the crescent-, IV- and MV-membrane. When expressed in vitro D13 readily forms trimers; under low salt conditions a higher-order structure resembling a honey-comb-like lattice can be observed but its structure is too heterogeneous to be analyzed in detail^9^. Indeed, one study suggested that in infected cells lattice-formation requires the synthesis of viral late proteins^10^. Conditional lethal mutants of VACV provide significant insight into the role of viral proteins in its replicative cycle, assembly and membrane biogenesis^2^. Five VACV proteins, the gene products of A6, A11, H7, L2 and A30, 5, collectively called virus membrane associated proteins (VMAPs), seem required for viral membrane biogenesis and the formation of infectious virus. We recently proposed, that the VMAP A11 is required for formation of the 50 nm vesicles, that are recruited to the D13 honey comb lattice to mediate viral membrane assembly. In the absence of A11, D13 accumulates in confined areas that are surrounded by closed ER-cisternae^11^; vesicle-formation and membrane opening seemed impaired, as assessed by EM-immuno-labeling and ET. Crescent-, IV- and MV-formation was absent, supporting our model that vesicle formation and membrane opening are necessary for VACV membrane-biogenesis^6^.

When the expression of H7, another VMAP, is blocked, IV- and MV-formation is absent as well^12^ arguing for an important role of this protein in virus assembly. The H7 sequence predicts a 17 kDa protein that by X-ray crystallography was shown to consist of seven α-helical domains, three β-strands and a 29 amino acids long C-terminal flexible tail. The seventh α-helical domain predicted a putative PX domain, involved in binding to phosphoinositides. Indeed, in dot blot experiments H7 was shown to bind to both PI3P and PI4P^13^.

The present study was conducted to further investigate the role of H7 in VACV-assembly. We confirm that H7 is required for IV- and MV-formation in HeLa cells. Surprisingly, we find that in the absence of H7 D13 collects in structures, that are significantly different from its honey-comb like arrangement, and that are intimately associated with the ER. Our data argue for a role of H7 in D13-lattice formation, thus shedding light on the molecular requirement for their formation.

## Results

### Thin section EM confirms that assembly is blocked in the absence of H7

When H7 is not synthesized the formation of IVs and MVs is blocked; dense inclusions accumulate collecting viral core proteins that are coated with structures resembling short viral crescents. A second structure accumulates close by, that collects D13^12^.

**Figure 1:**
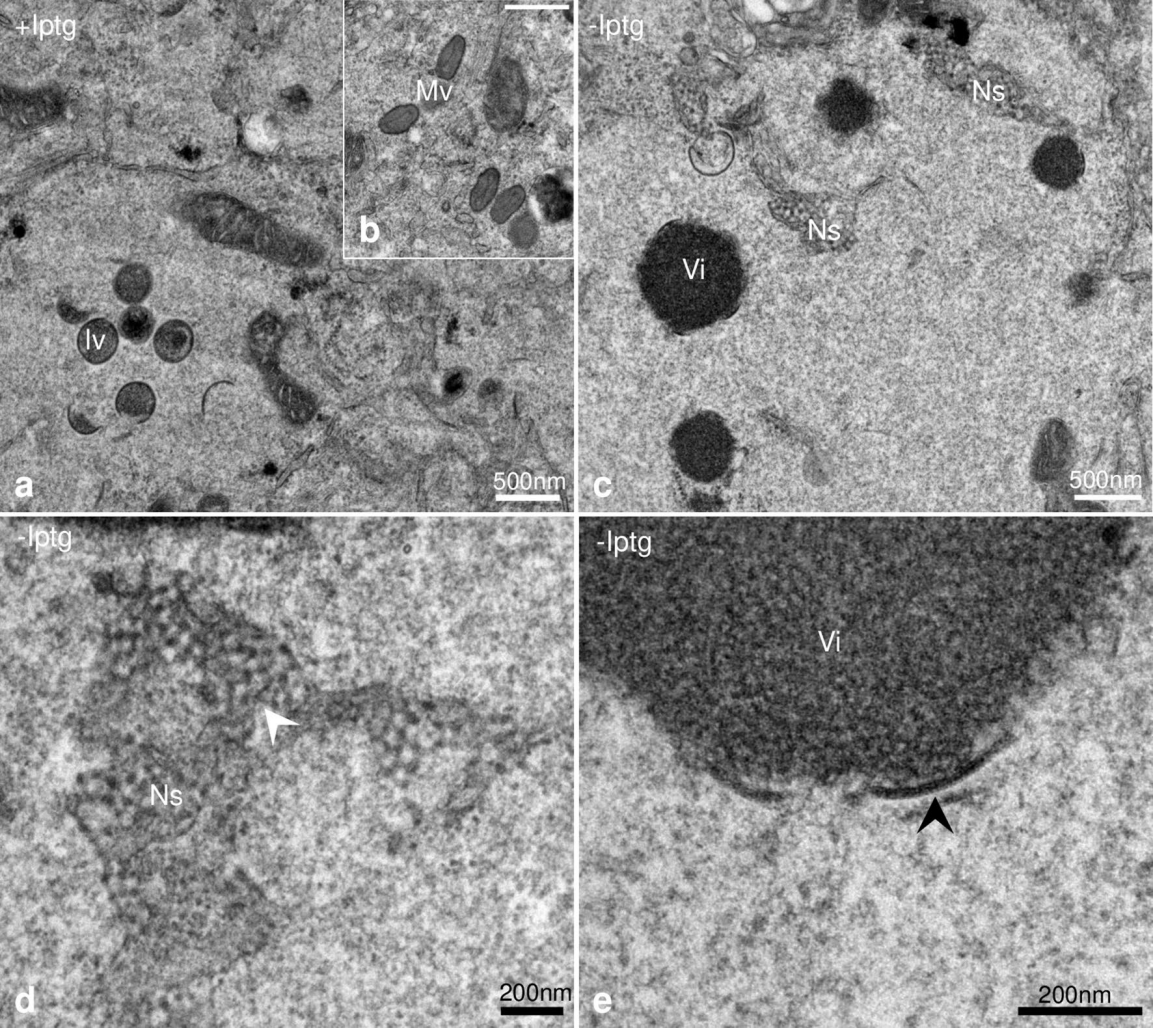
In the absence of H7 two prominent structures accumulate in infected HeLa cells. HeLa cells were infected and fixed 12 h post infection, embedded in epoxy resin, sectioned and imaged by TEM. Infected cells with IPTG (+ Iptg; **a**,**b**) show wild-type phenotype with immature virus (Iv) and mature virus (Mv). Without IPTG (-Iptg) infected cells display two prominent structures (**c**): network-like structures (Ns) and electron dense areas collecting core protein, virosomes (Vi). At higher magnification the NS is composed of electron-dense dots arranged in a regular pattern (**d**; white arrowhead). The electron dense area (virosome, Vi) in (**e**) displays short crescents-like structures (black arrowhead).

Conventional EM of HeLa cells fixed at 12 h post-infection largely confirmed these observations. In the presence of isopropyl-b-D-thiogalactopyranoside (IPTG), structures typical of a wild-type infection (crescents, IVs and MVs) were observed (Fig. 1a,b). Without H7-synthesis IVs and MVs were absent; large areas of lower electron-density, the VACV-replication site, collected electron-dense aggregates (Fig. 1c; Vi) that were occasionally coated with arcs resembling short crescents (Fig. 1e). At the periphery of the replication sites a second prominent structure, absent in the presence of IPTG, was seen. The accumulation of this second structure was observed before but our EM-embedding method showed these to consist of electron-dense spots seemingly arranged in a regular pattern and associated with membranes reminiscent of the ER (Fig. 1d; Ns).

**Figure 2:**
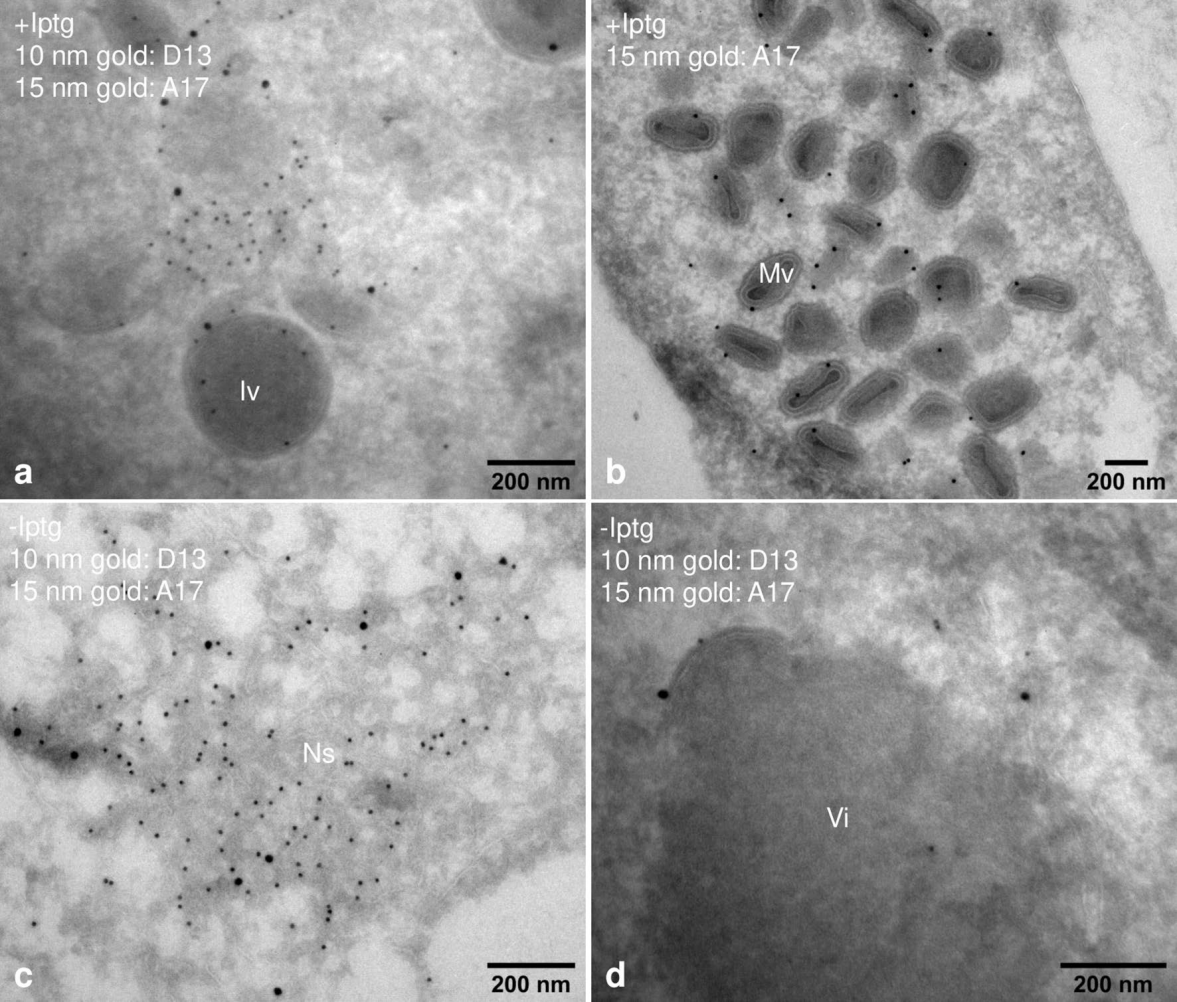
EM-immunolabeling shows the accumulation of D13 on the network structure. HeLa cells were infected as for Fig. 1 and embedded for EM-immuno-labeling. With IPTG (+ Iptg) immature virus (Iv) and mature virus (Mv) show labeling for A17 and D13 (**a**,**b**). (**c**) Double-labeling of the Ns that accumulates without IPTG (-Iptg), showing abundant labeling for D13 and labeling for A17 on membranes, indicating the presence of the ER. The short crescents that accumulate on the electron-dense structures (Vi) in (**d**) are labeled by D13 and A17 (**d**).

EM immuno-labeling confirmed the expected localization of VACV proteins in the presence of IPTG; anti-D13 to the surface of the IV membrane (Fig. 2a) and anti-A17 to the membrane of both the IVs (Fig. 2a) and the MVs (Fig. 2b). The electron-dense aggregates in the absence of IPTG collected the core protein A3 (not shown) while the short arcs were labeled with anti-D13 and A17 (Fig. 2d), confirming that they are short crescent-membranes. The network labeled prominently for anti-D13 confirming that the scaffold protein accumulated in these aberrant structures. Anti-PDI confirmed that the membranes were derived from the ER (not shown). A17, the binding partner of D13, localized to the surrounding ER membranes (Fig. 2c) where it was not particularly enriched when compared to random pieces of ER (not shown).

The collective data suggested that H7 was involved in the proper formation of D13 in infected cells, resulting in the dense network associated with the ER. The D13 structures were next analyzed in 3D by ET.

### Scanning- and transmission electron tomography reveal an aberrant organization of D13

**Figure 3:**
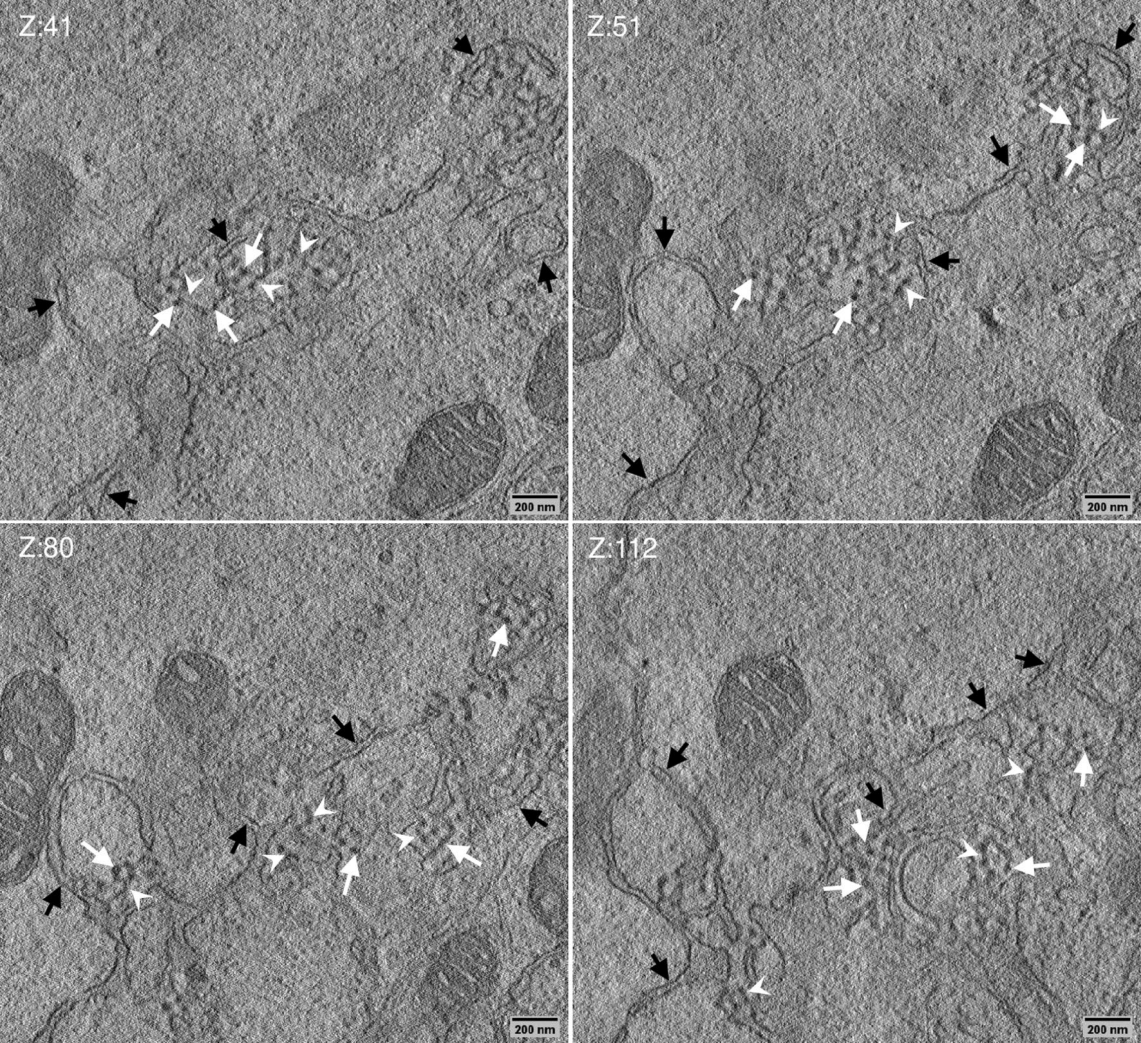
The D13 network structure is intimately associated with the ER. HeLa cells infected without IPTG were prepared as in Fig. 1. Sections, 750 nm in thickness, were subjected to STEM tomography. The figure displays four slices of the reconstructed tomogram slice 41, 51, 80 and 112 (Z:41, Z:51, Z:80 and Z:112). It shows the electron-dense spots (white arrows) displaying a regular pattern. Slice by slice inspection also shows that the spots are connected by thin structures, likely thin membrane tubes (white arrow head; Movie S1). The network structure is associated with, and surrounded by, the ER (black arrows). The slices are taken from the supplemental Movie S1.

**Figure 4:**
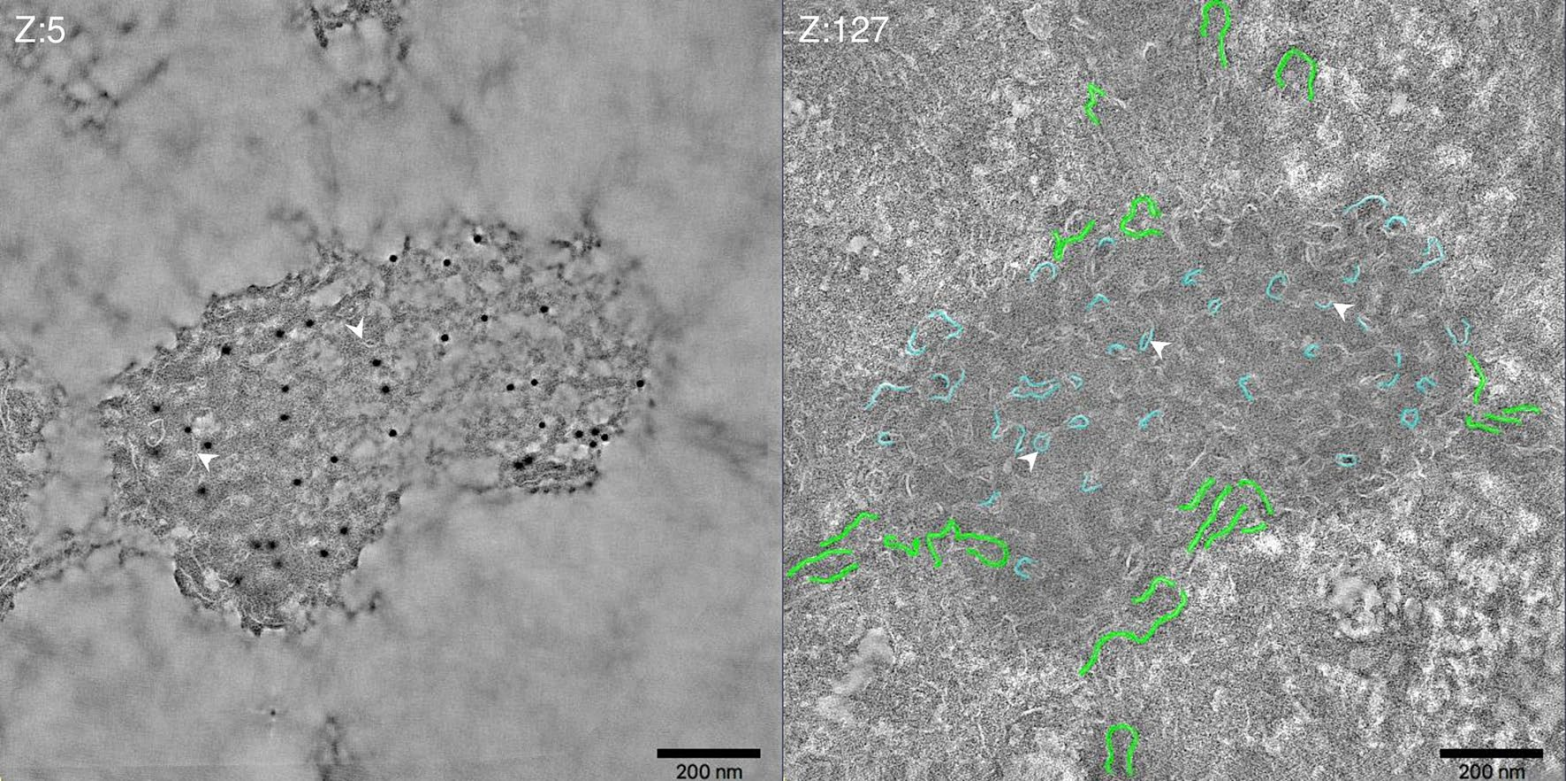
Short membrane tubes prominently accumulate on the D13 network. HeLa cells infected without IPTG were prepared as for Fig. 2. Sections, 150 nm in thickness, were labeled with anti-D13 and subjected to dual-axis tilt series acquisition by transmission EM. Two slices of the reconstructed tomogram; the surface of the section (Z:5) displays anti-D13 labeling and identifies the D13-positive network structure in Tokuyasu sections. The slice Z:127 illustrates that the D13 network is filled with short membrane tubes (white arrowheads) that appear as white lines due to the negative contrasting in the Tokuyasu method. A subset of the short membrane tubes is rendered in light blue, while the ER cisternae that accumulate close by are rendered in green. The slices are taken from the supplemental Movie S4.

Sections, 750 nm in thickness, of conventionally embedded samples were analyzed by scanning transmission electron tomography (STEM-ET), focusing on the 3D organization of the D13-network structure. In 3D the intimate association of the D13-network with the ER was readily observed (Fig. 3; Movies S1 and S2). The rendering shown in Movie S2 illustrated how the ER cisternae surrounded, and moved perpendicular into, the network (Fig. 3; Movie S2). Slice by slice inspection of the tomograms revealed that the spots were connected to each other by thin tubes of distinct length, altogether contributing to the regular network appearance (Fig. 3 white arrowheads; Movie S3). The electron-dense spots were seen to pull on the ER membrane to form a tube, suggesting that the membrane tubes were derived from the ER-membrane (Fig. 3; see also below). Rendering of the electron-dense D13-spots in z, within the 750 nm volume of the section, displayed them as short hollow tubes; within the limitation of the EM-embedding and contrasting used these measured roughly 22 nm in width (Movies S2 and S3). Dual-axis transmission electron tomography of thawed cryo-sections, 150 nm in thickness, labeled with anti-D13 confirmed first that the scaffold protein accumulated within the network (Fig. 4 Z:5). Second, the D13-labeled structures showed a massive accumulation of short membrane fragments (Fig. 4 Z:127), that appeared as white lines due to the negative contrasting used in this method (Fig. 4, Movie S4). Their abundance strongly suggested these to correspond to the membrane tubes connecting the D13 units shown by STEM-ET. The electron-dense D13-spots were not readily visible in the tomograms of the thawed cryo-sections, likely because of the negative contrasting and the smaller volume analyzed.

The collective 3D-EM data argued that in the absence of H7, D13 forms a dense 3D network probably made by interconnected by short membrane elements, forming a regular pattern, intimately associated with ER-cisternae. The structure of the D13-spots was next analyzed with a hybrid cryoET method.

### Refrozen Tokuyasu sections show D13 trimers but a failure to form hexagons

Under wild-type infection conditions D13 forms hexamers of D13-trimers. The trimers measure 7–9 nm in diameter, while the hexamers measure roughly 20–22 nm from vertex to vertex, depending on the EM method used^4,6^. The average width of 22 nm of the D13-spots, measured in the STEM-tomograms, suggested that D13 might form hexamers but that lattice formation was impaired in the absence of H7. However, the conventional embedding and contrasting used, displayed D13 as uniform electron-dense dots and failed to resolve trimers or hexamers.

**Figure 5:**
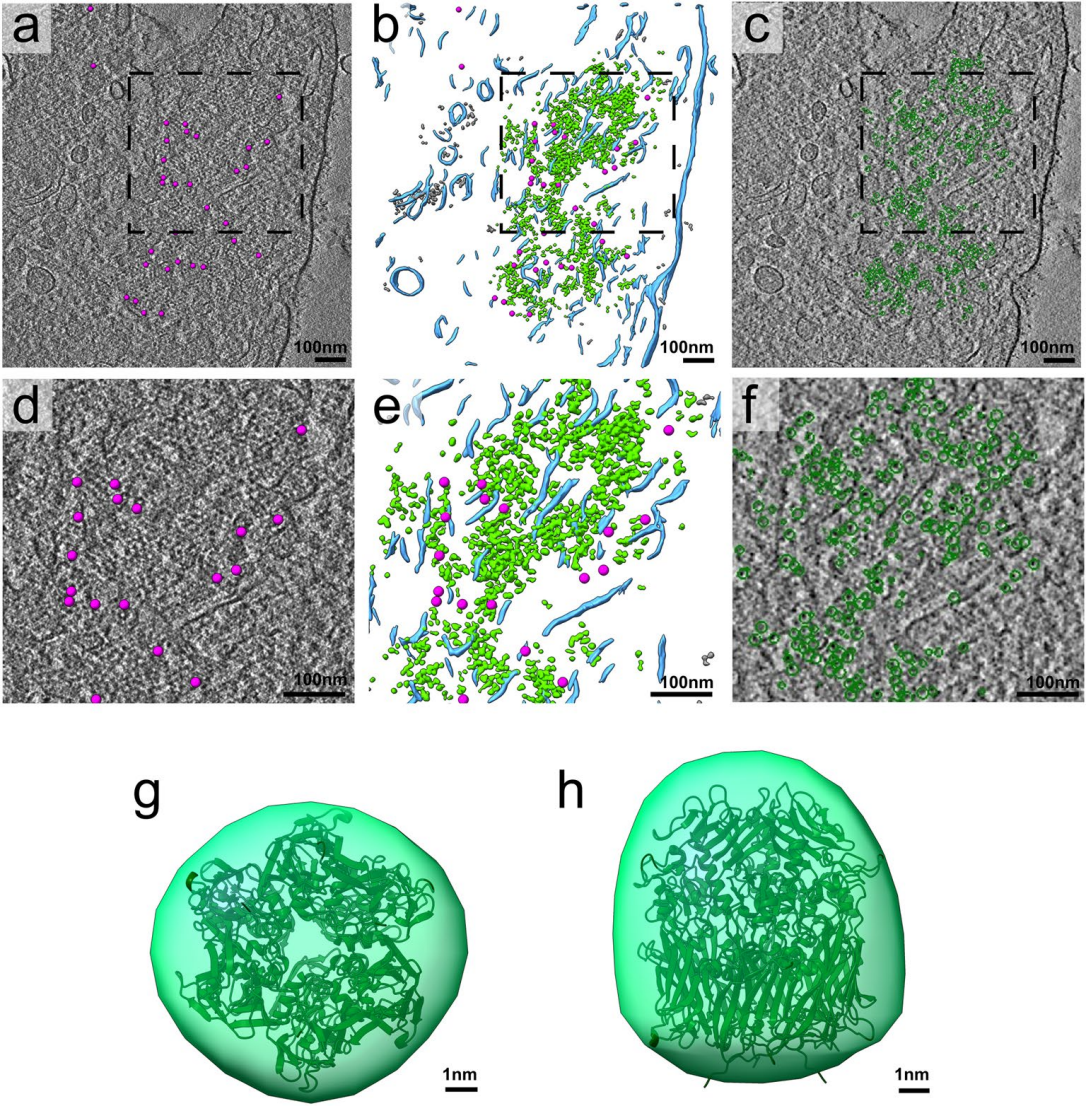
D13 trimers fail to form hexamers the absence of H7 synthesis and clusters together with membranes. HeLa cells infected for 12 h with VACVindH7 without IPTG (**a**–**f**) were processed for cryo-sectioning, thawed cryo-sections immuno-labeled with anti-D13 and protein A coupled to 10 nm gold, followed by plunge freezing. In (**a**), a single slice of the tomographic volume is shown (slice 43, voxel size = 12.67A), the gold beads localized in the initial slices of the volume have been marked on top using magenta spheres of 10 nm in size. In (**b**) the tomographic volume annotations are presented as follows: membranes are shown in light blue, gold beads in magenta, D13 trimers in green. Densities recognized along the D13 trimeric particles by the application of the CNN trained algorithm that are located outside the D13 cluster are displayed in grey. In (**c**) the slice number 50 of the same volume is displayed. Here the green circles indicate the particles that are picked for the generation of the initial averaged model. In (**d**–**f**) a zoom of the respective boxed areas of (**a**–**c**). In (**g**,**h**) two perpendicular views (bottom and side view) of the initial D13 model in green superimposed to the crystal structure of the D13 trimer (dark green ribbons, PDB id 2YGB).

The technique of refrozen Tokuyasu sections^14^ was applied to analyse the D13-organization in the absence of H7-synthesis. Cryo-sections, 70nm in thickness, were thawed and labeled with anti-D13, the sections were vitrified by plunge-freezing and imaged by cryoET. In the presence of IPTG the honey-comb lattices were readily observed in 3D, located on the surface of the IVs or as small discrete patches next to the IVs, as shown before (supplemental Figs. S3A–C, Movie S5;^6^). By inverting the contrast, the organization of D13 trimers into hexamers, together forming the honey comb lattice was apparent (supplemental Figs. S3B,C, S4A–F, Movie S5). In the absence of IPTG the D13-network could not be unequivocally identified without prior immuno-labeling in refrozen sections as shown above. Areas labeled for D13 on the surface of the section were subjected to tilt-series-acquisition and structures analyzed by sub-tomogram averaging (Movie S6). Structures with an average diameter of 8-9 nm, were readily observed in these areas (Fig. 5a–f). The area that labeled positive for D13 contained clusters of 8-9 nm wide structures interspaced by short membranes (Fig. 5a–f). By extracting particles from this area, we were able to produce an initial low resolution averaged model with a general shape and size approaching the one of the D13 trimer as superposition to the known trimeric crystal structure of the D13^8^ showed (Fig. 5g,h). Although the trimers typically clustered in specific areas, we failed to observe the typical hexagonal arrangement of the honeycomb lattice (Fig. 5a–f, supplemental Fig. S3D–F).

We propose that the electron-dense spots seen by RT-EM are composed of D13-trimers, lacking the hexagonal arrangement. The clusters of trimers are intimately associated with short membrane tubes as shown both by STEM-ET and cryoET. The analysis of larger volumes (STEM-ET), suggests that the D13 trimers may form tubes interconnected by short membrane-elements.

### Localization of H7

A putative role for H7 in proper formation of the D13 into honey-comb patches was surprising and prompted us to re-investigate its localization in infected cells, previously proposed to be predominantly cytoplasmic^12^. We took advantage of the fact that recombinant H7-protein expressed in the presence of IPTG was tagged with an HA-epitope. In addition, infection with VACV-H7ind without IPTG and transfected with HA-tagged full-length H7 was used to over-express H7 using a VACV synthetic early/late promotor. Transfection efficiency was roughly 90%, based on anti-HA labeling by light microscopy. Expression of the full-length H7 lead to an efficient rescue of the phenotype observed without H7-synthesis, showing that transfected HA-H7 was functional (supplemental Fig. S2A). HA-tagged H7 expressed in the presence of IPTG or upon transfection displayed a general cytoplasmic labeling both by LM (supplemental Fig. S1) and EM (data not shown) with no concentration on, or close to, the viral membranes. While surprising, they confirm previous results^12^.

### Residues required for assembly

Expression of HA-tagged H7 in trans produced a phenotype indistinguishable from infection in the presence of IPTG; infected/transfected cells displayed the full complement of crescents, IVs and MVs, while the electron-dense virosomes and ER-associated networks were absent (supplemental Fig. S2A; Table 1). The efficient rescue of the H7-phenotype prompted us to analyse residues required for membrane assembly. While single residues within H7 required for infectivity were previously analysed^13^, we focused on rescue of assembly scoring for IV and MV-formation by EM. In first instance we used truncated constructs of H7, expressing amino acid 1–114 (N-terminus) or 119–146 (C-terminus) of H7. Both constructs failed to rescue assembly, implying that both the N- and C-terminal part of the protein is essential for D13 organization (Table 1).

**Table 1:** Ability of various mutant H7 proteins to rescue the H7-phenotype.

H7-expression construct	IV	MV	Virosome	Network
VACV H7ind + IPTG	+	+	−	−
VACV H7ind-IPTG	−	−	+	+
pEL-HA-H7	+	+	−	−
pEL-HA-H7-R109E	+	+	−	−
pEL-HA-H7-R117E/K128E	+	+/−	+/−	−
pEL-HA-H7-K108E/R109E/K112E	−	−	+	+
pEL-HA-H7-119-146	−	−	+	+
pEL-HA-H7-1-114	−	−	+	+

HeLa cells were infected with or without IPTG as positive and negative control, respectively. Cells infected without IPTG were transfected one hour after infection with the respective constructs where synthesis of the H7 constructs is driven by a synthetic early/late VACV promotor (pEL) and the expressed protein tagged with HA. Transfection efficiency was assessed by parallel fluorescent labeling using anti-HA and estimated to be roughly 90% in all experiments. Cell profiles were inspected for presence or not of viral forms, IVs, MVs, electron-dense virosomes or network structures (the latter two typically only appear when H7 is not synthesized or not functional). The full length (pEL-HA-H7) construct rescues the formation of IVs and MVs and virosomes and network structures are absent. All single point mutations also rescue IV and MV-formation and the results for the R109E mutant are shown as example.

Previous experiments implied an important role for positively charged amino acids of the H7 protein for infectivity^13^. Specifically, substituting the lysins at positions 108, 128 and 143 individually, affected the production of infectious virus, as well as a triple mutant substituting lysine 108, arginine 109 and lysine 112. The latter three amino acids map in the putative PX, PIP-binding domain of H7. We substituted all positively charged amino acids individually, expressed the mutant proteins as described above and quantified the different viral forms, IVs and MVs (Table 1). None of the single point mutations affected assembly and the full complement of viral forms was made to the same extend in transfected cells (Table 1; supplemental Fig. S2B and data not shown). Mutating all three positively charged amino acids in helical domain 7 failed to rescue assembly (Table 1; supplemental Fig. S2d). In addition, a double mutant substituting lysine 128 and 143 also affected assembly, IVs were observed, whereas MVs were absent (Table 1; supplemental Fig. S2C).

The data thus show, and confirm, an important role for the helical domain 7, in particular its three positively charges amino acids, its putative phosphoinositide binding domain.

## Discussion

Although biogenesis of the VACV-membrane remains controversial until this date, viral proteins involved have been identified thanks to conditional deletion mutants^2^. A recent focus is a set of less abundant viral proteins, the VMAPs, that are important for viral membrane assembly and thus the production of infectious progeny. Thus, we recently analyzed the role of A11 by 3D EM; in its absence D13 collects in cytoplasmic patches, seemingly with a honey comb arrangement, surrounded by ER-cisternae^11^. We concluded that A11 might be involved in the formation of the 50 nm vesicles, which we predict in our model to be the precursors of VACV membrane biogenesis, and in their membrane rupture. The present study asked whether the gene product of H7, a 17 kDa VMAP, could also mediate membrane rupture. In its absence short crescents with open ends are formed that are associated with viral core proteins suggesting a role in membrane elongation rather than rupture. This discriminates H7 from A11 where crescent formation and membrane rupture is absent altogether. Our data suggest that without H7, D13 is able to form trimers, but the formation of hexamers and the high order honey comb-structure, is impaired. This places H7 into the first viral factor required for D13 organization in infected cells and shows that this organization is essential for proper viral membrane assembly.

In the absence of H7 D13 forms clusters that are intimately associated with the ER, showing a high affinity of D13 for these membranes. The latter are not particularly enriched for A17, the membrane protein proposed to mediate membrane binding of D13. This observation is similar to the phenotype seen in the absence of A11 expression, where D13 accumulations are surrounded by ER-cisternae that are not enriched in A17^11^. Our STEM ET data show that the D13 structures formed are connected by short membrane tubes forming a regular network-like structure. The overall organization of the network was not readily observed in cryoET because of the much smaller volume analysed (750 nm versus 70 nm in z), exemplifying the strength of STEM ET. D13 protein assemblies composed of several trimers, formed hollow tubes that, in turn, were interconnected by thin membrane tubes with a regular shape and length. It suggests that D13 has an intrinsic ability to recruit, bind to and modify membranes of the ER, although at present is it not clear if D13 is the sole factor mediating this.

With an important role in both membrane elongation and D13 organization, the cytoplasmic localization of H7, without co-localization with viral structures, is surprising. The lipidome of VACV shows an enrichment for phosphoinositides (PIs^15^). A possible scenario is that H7 transiently binds via its PX-domain to a specific PI located to the viral growing membrane, mediating its elongation. H7 also seems to play a role in D13 lattice-formation, promoting hexamer formation and likely trimer/trimer interactions. Failure to localize H7 to the D13-lattice could indicate that its HA-tag is inaccessible to the antibody used or that it is a minor constituent of the viral honey-comb lattice. Indeed, the capsid proteins of other viruses of the giant virus-family, that show structural homology to D13 and also arrange in hexagonal arrays on the surface of mature virions, may be stabilized by minor viral proteins (reviewed in^16^). A final scenario is that proper organization (in a honey-comb like lattice) of membrane-associated D13 mediated by H7 facilitates membrane elongation. Short crescents are produced with a proper D13 lattice that fail to grow since the majority of D13 is mis-organized and not available for membrane biogenesis. Experiments are currently carried out to test these hypotheses. However, due to its non-co-localization with typical viral structures involved in VACV-assembly we did not attempt to analyse H7 interacting partners.

Altogether, H7 plays an important role in the cytoplasmic organization of D13 that we show to be essential for proper membrane assembly of VACV. As small crescents are formed, H7 is not required for membrane rupture perse. For the latter A11 is a more likely candidate as no open membranes are observed in its absence. Finally, our data also suggest an intrinsic affinity of D13 for membranes of the ER, not necessarily involving its binding partner A17. This affinity may lead to a reorganization of the ER-membrane that, in the absence of H7, results in the formation of thin membrane tubes and, we speculate, in the recruitment of 50 nm vesicles under wild-type infection conditions. Further studies are now on the way to elucidate the role of other VMAPs, their role in D13 organization and membrane biogenesis.

## Materials and methods

### Cell and virus culture

HeLa cells (ATTC-CCL-2) and BSC-40 cells (a kind gift of Jason Mercer, university of Birmingham, UK) were grown in Dulbecco’s modified Eagle’s medium (DMEM) containing penicillin and streptomycin and 10% fetal calf serum. To prepare VACV-H7-ind, a kind gift of Bernie Moss^12^, BSC-40 cells were grown to 70% confluency. Cells were infected in the presence of 100 μm isopropyl-β-D-thiogalactopyranoside (IPTG; Sigma) at low MOI (0.1) and virus harvested 3 days post-infection as described^17^. Virus titers were determined by light microscopy by infecting HeLa cells with twofold dilutions of concentrated virus in the presence of IPTG and fixed 8 h post-infection. The percentage of infected cells was estimated by indirect immunofluorescence using an antibody to A14.

### Antibodies

Antibodies used are described in^11^. In brief: anti-A14^18^, anti-core^19^, anti-D13^20^, anti-A17 (raised to amino acid 17–37 of A17;^21^) and anti-HA (Sigma). Protein A couple to 10 or 15 nm gold was purchased from central microscopy core facility (CMC) Utrecht, the Netherlands.

### Sample preparation for EM and ET

Infected cells were processed for EM-immuno-labeling as described in^11^. In brief, at the indicated time post-infection cells were fixed for 1 h at room temperature (RT) in 4% paraformaldehyde (PFA; electron microscopy sciences cat# 15710) and 0.1% glutaraldehyde (GA; electron microscopy sciences cat#16216) in PHEM buffer (60 mM Pipes, 25 mM HEPES, 10 mM EGTA, 2 mM MgCl_2_, pH 6.9) and kept at 4 °C in 4% PFA in PHEM buffer until further processing. For Tokuyasu cryo-sectioning, thawed cryo-sections were single- or double-labeled as described^22^. Labeled sections were contrasted and dried in 0.4% uranyl acetate in 2% methylcellulose. For Epoxy resin embedding all reagent were from Sigma; the fixed cells were, post-fixed with 2.5% GA, pelleted and mixed 1:1 with 3% agarose. The agarose was cut and the cubes post-fixed with 1% OsO_4_, followed by contrasting with 0.1% (w/v) tannic acid in 0.5% HEPES, pH7.4. After washing twice with 1% Na_2_SO_4_ and three times with water, the cubes were contrasted with 2% UA in water, followed by dehydration with increasing concentration of ethanol. Dehydrated sample was infiltrated with increasing concentration of epoxy resin diluted in ethanol, before infiltration with pure resin and polymerization at 65 °C.

### Transmission electron microscopy, (cryo-) electron tomography

Thin (70 nm) immuno-labeled sections were observed in a Jeol 1400 Flash operated at 80 kV and equipped with a Xarosa camera. Dual-axis tilt series of thawed cryo-sections 150 nm in thickness labeled for anti-D13 were acquired with a Tecnai (Thermofisher) operated at 200 kV with field emission gun using a Fishione dual-axis holder, and equipped with a Gatan Ultrascan 4000 camera. Briefly, grids were mapped using SerialEM and positions of interest stored for semi-automated image acquisition of tilt series increment 1, binning 2 at 19,000 magnification (pixel size 0.37 nm) at tilt angles ± 70°. For cryoET of vitrified sections, 70 nm cryo-sections were placed on holy carbon coated grids (Quantifoil R 2/2; EM services), thawed and immuno-labeled. Sections were washed with PHEM buffer and after brief blotting (3 s) plunge frozen using EMGP (Leica) and 2 s for post-blotting time. Cryo-tomograms were acquired with a Tecnai F20 TEM operating at 200 kV with SerialEM using low-dose mode^23^. The Gatan 626 cryo-holder was used at tilt angles − 46 to + 52 degrees, increment 2 at a magnification of 29 K (no binning -pixel size 0.3168 nm using the US4000 CCD Gatan camera). WBP was used with the SIRT like filter of 7 iterations. For STEM tomography, 800 nm sections of resin-embedded cells were cut and placed on bar grids without formvar coating. Gold particles were placed on both sides of the sections and overlayed with a thin layer of carbon. STEM tilt series were acquired with a Jeol JEM-2100F at 200 kV and the tilt angles − 72° to + 72° with 1.5° increment. The tilt series were recorded with the EM-Tools software (TVIPS, Tietz) and each image had 1024 × 1024 with the pixel size 2.74 nm. All tilt series were reconstructed in IMOD using protein A coupled to 10 nm gold as fiducials^24^. Movies and rendering were made in IMOD and Fiji.

### Quantification

Cryo-tomograms were binned 4 times using the binvol function of IMOD and the Mitchell antialiasing filter before further analysis. Smaller volumes were extracted, and their contrast was inverted using the mrcbyte function of IMOD with the -R flag on, prior to proceed with ChimeraX^25,26^ iso-surface representations (Fig. 5f) or ImageJ/Fiji^27^. In the case of infection with IPTG, the scale space filter^28^ was used on top to have the honeycomb lattice easily observable in close zoom. Mean lattice distances were calculated by tracing an IMOD model (Fig. 5c) and extract their lengths through the imodinfo function. Fiji was used to assay the Delaunay Voronoi mean distance after manual picking of positions of the D13 trimer in the honeycomb lattice of the wild type VACV scaffold and the D13 clusters observed at the H7 mutant (Supplemental Figs. S3 and S4)^27^.

### Volume annotation, particle picking and initial model generation

For the volume annotation, three training sets, one per feature, were prepared to train the convolutional neural network (CNN) algorithm implemented in eman2.9^29^. These training sets were based on the following features: membranes, D13 trimers and gold beads and were prepared based on positive manually segmented examples and negative areas missing the targeted particles. After training the network, the algorithm was applied to the entire tomographic volume. Final segmentation/annotation results were visualized as iso-surfaces with UCSF ChimeraX. False positives were removed by thresholding out smaller sized particles for each feature using the ‘Hide Dust’ tool of Chimera. For particle picking of D13 trimers the annotation applied in the previous step was used. Coordinates of 2000 particles were extracted (box size of 16 × 16 × 16) based on the annotated volume and inspection of the results followed. From them around 500 particles removed manually based on the following criteria: (a) located outside the D13 cluster and (b) falsely picked membranes inside the cluster instead of D13 particles. The rest of the remaining particles were used for the generation of the initial model using the stochastic gradient descent algorithm of eman2.9^30^ applying a threefold symmetry axis. The D13 trimer crystal structure was fitted to the low-resolution resulting model of D13 in ChimeraX (Fig. 5g,h).

### Cloning, transfections and light microscopy

For transfection experiments, constructs of HA-tagged wild-type or mutants H7 were used. H7 gene was amplified by PCR using vaccinia virus genomic DNA as template. PCR product was then digested with NotI and SacI and subcloned into the modified pBluescript II vector^31^. The same protocol was used for mutant’s constructs using strings DNA fragments (Thermofisher) as template for PCR. All constructs were validated by sequencing. HeLa cells grown on glass slides were infected with VACV H7ind in the presence of 100 μM IPTG and fixed at 12 h post-infection with 3% PFA. Cells were permeabilized with TX-100 and triple-labeled with anti-A17, anti-HA and DAPI followed by confocal microscopy. For transfection, 1 × 10^5^ cells were seeded per well of a 24-well plate. Cells were infected with VACV H7ind without IPTG for one hours at 37 °C. Meanwhile the transfection mix was prepared in Opti-mem (Gibco), according to the instructions of the manufacturer using 1 μg of plasmid DNA and 1 μl of Lipofectamine 2000 (Invitrogen) per well and the mix incubated for 15–30 min at RT. After infection cells were washed with serum-free medium and the transfection mix added dropwise to the cells. After incubation for another 4 h at 37 °C, the transfection mix was removed and replaced by serum-free medium. Cells were fixed at the indicated times post-infection and processes for conventional EM or for light microscopy.

## Supplementary Information

Supplementary Video 1.

Supplementary Video 2.

Supplementary Video 3.

Supplementary Video 4.

Supplementary Video 5.

Supplementary Video 6.

Supplementary Information 1.

Supplementary Figures.

## Data Availability

All data generated or analysed during this study are included in this published article and its supplementary information files.
