# Supplementary material for: Vaccinia virus H7-protein is required for the organization of the viral scaffold protein into hexamers
Source: Sci Rep. 2022 Jul 29;12:13007. doi: 10.1038/s41598-022-16999-2 (PMC9338303; doi:10.1038/s41598-022-16999-2)
Supplement: Supplementary file 7 — Supplementary Information 1. [file 41598_2022_16999_MOESM7_ESM.docx]

**Supplement**

1. **Movie 1 (figure 3). STEM-ET of VACV H7ind without IPTG**

HeLa cells were infected, fixed and embedded as described for figure 1. 750nm section placed on bar grids with gold particles placed on both sides of the grid were subjected to tilt series acquisition in STEM mode from +72 to -72 degrees.

1. **Movie 2 (figure 3). Rendering of the STEM tomogram of movie 1.** The ER-green, D13

spots in red. It shows the massive accumulation of ER cisternae around the D13 structures that wrap around them and seem to move perpendicular into them.

1. **Movie 3 (figure 3) higher magnification view of a part of movie 1 rendered.** Green-

ER, red-D13 spots and light blue thin membrane tubes that connect the D13 structures

1. **Movie 4 (figure 4). Dual-axis ET of thawed cryo-sections labeled with anti-D13.**

250 nm sections of HeLa cells infected for 12 hrs with VACV H7ind without IPTG were immuno-labeled with anti-D13 and subjected to dual-axis tilt series acquisition from +70 to -70 degrees. The negative contrasting using in this method displays the membranes as white lines that are abundantly present as short cisternal segments located within the D13-positive structure.

1. **Movie 5 (figure 5A to C). CryoET of refrozen tokuyasu sections of HeLa cells infected with VACV H7ind with IPTG.**
2. **Movie 6 (figure 5D to F). CryoET of refrozen tokuyasu sections of HeLa cells infected with VACV H7ind without IPTG and labeled with anti-D13 prior to plunge freezing**
