# Supplementary material for: Vaccinia virus H7-protein is required for the organization of the viral scaffold protein into hexamers
Source: Sci Rep. 2022 Jul 29;12:13007. doi: 10.1038/s41598-022-16999-2 (PMC9338303; doi:10.1038/s41598-022-16999-2)
Supplement: Supplementary file 8 — Supplementary Figures. [file 41598_2022_16999_MOESM8_ESM.docx]

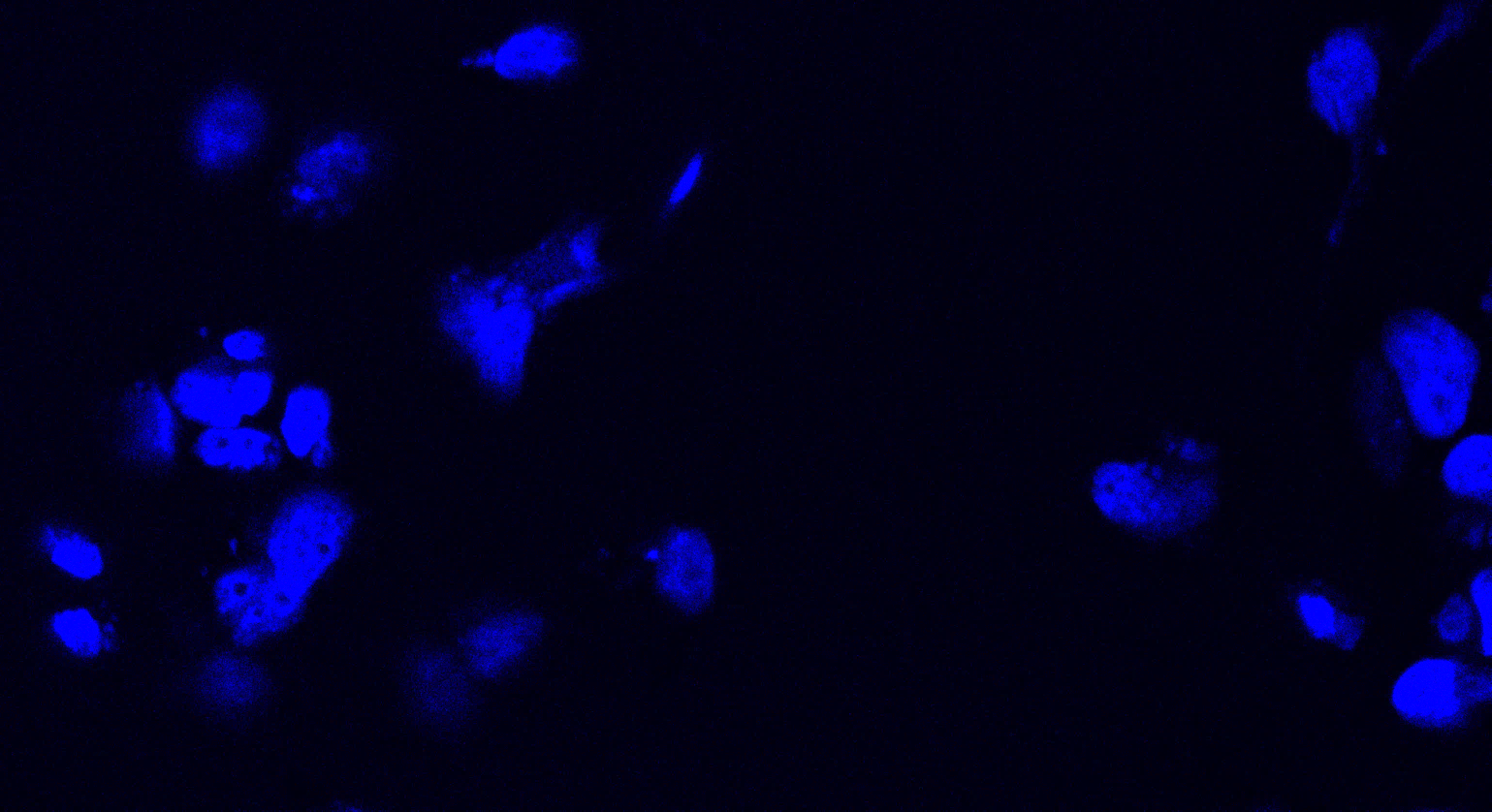

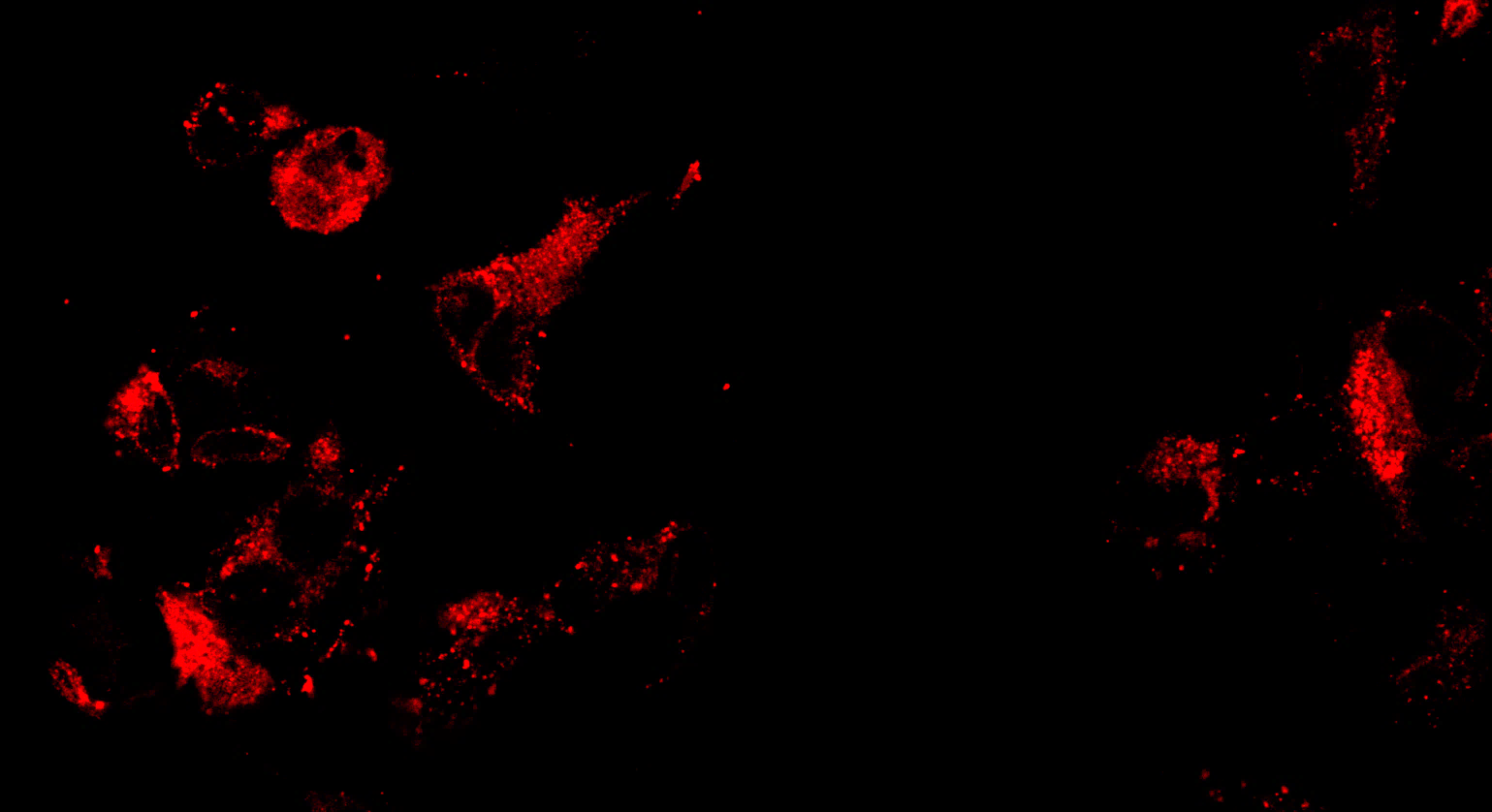

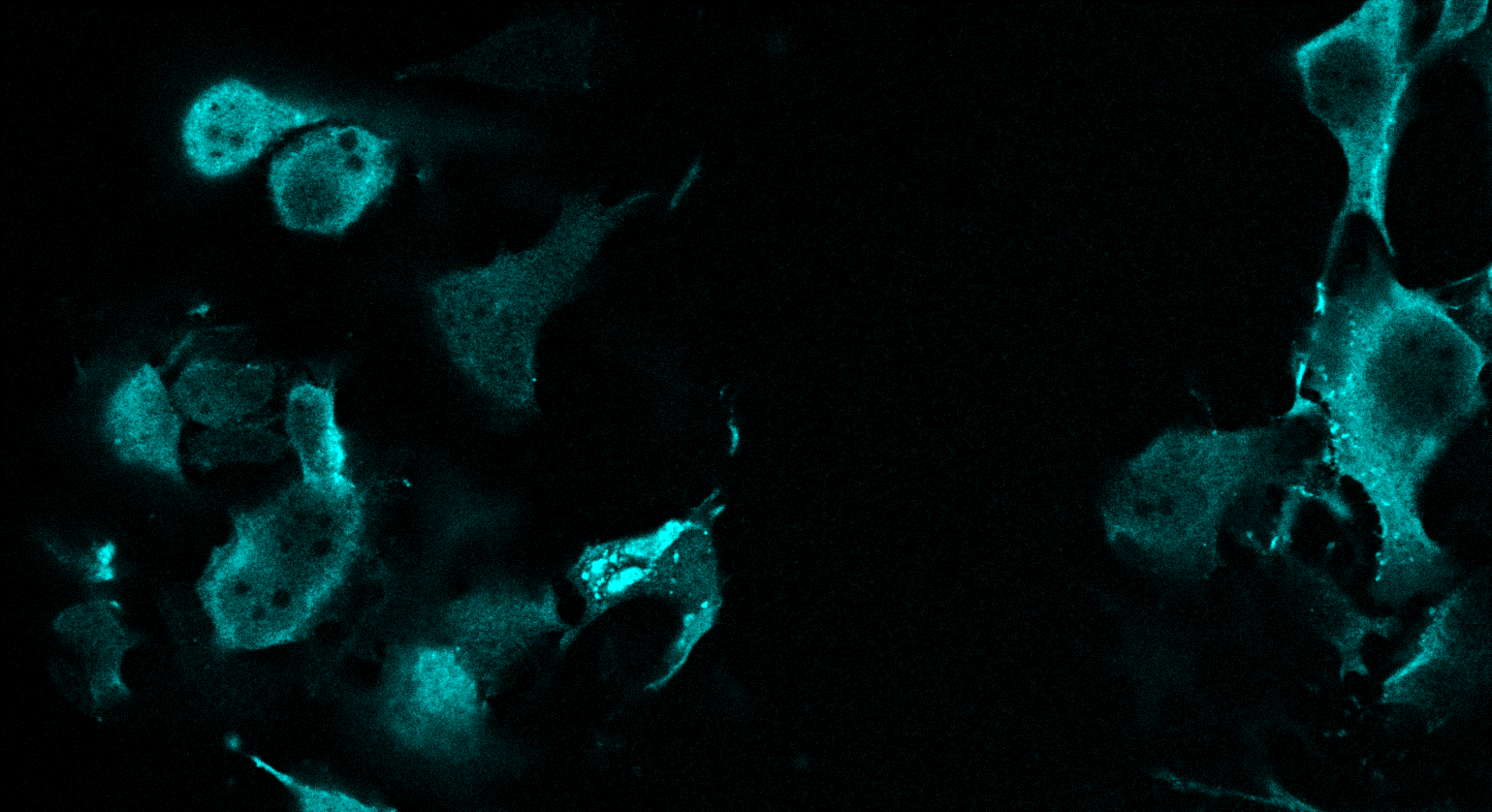

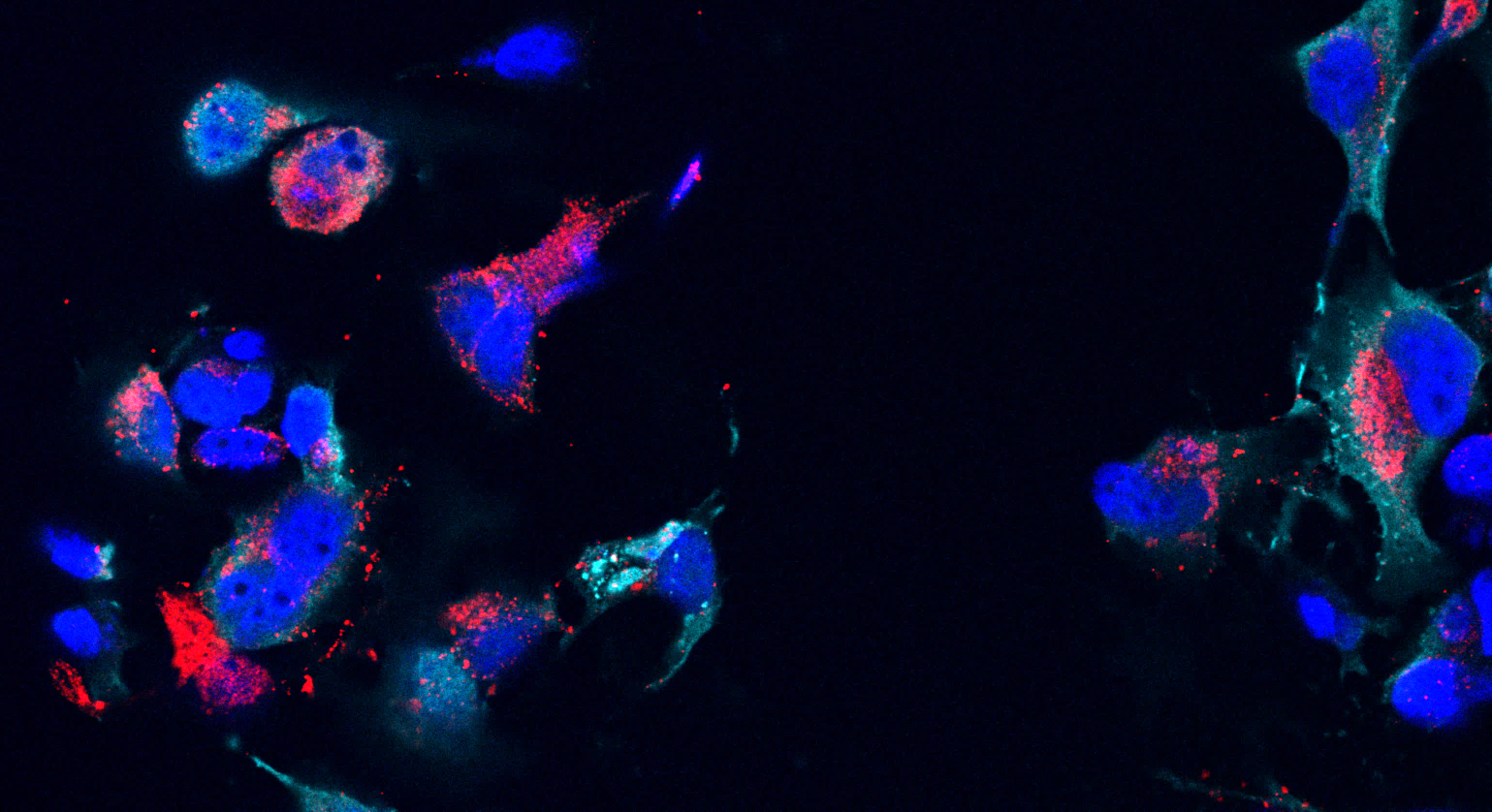


DNA

merge

H7

A17

**Supplemental figure 1: Localization of H7 by light microscopy**

HeLa cells were infected with VACV H7ind in the presence of 100μM IPTG and fixed at 12 hrs post-infection. Fixed cells were triple labeled with anti-A17, anti-HA (localizing H7 tagged with HA) and DAPI. A17 shows the typical punctate labeling co-localizing with the DAPI-positive replication sites in the cytoplasm. H7, localized using anti-HA, displays a cytoplasmic labeling with no co-localization with A17 or the replication sites.

**Supplemental figure 2: EM phenotypes observed after rescue with single, double and triple deletion mutants.**

HeLa cells were infected in the absence of IPTG and transfected one hour later with the indicated constructs. Transfection of full-length wild-type H7 (A) results in complete rescue displaying IVs and MVs. The single point mutant of H7, R109E, shown exemplary for all single point mutations, is also able to rescue wild-type infection (B). Mutating two positively charged amino acids R117E and K128E (C) results in partial rescue; MV formation is impaired and IVs accumulate instead. The triple mutant (D) K108E, R109E, K112E mutating the three positively charged amino acids in the 7^th^ helical domain fails to rescue IV- and MV-formation; instead virosomes (Vi) and network structures (NS) accumulate.


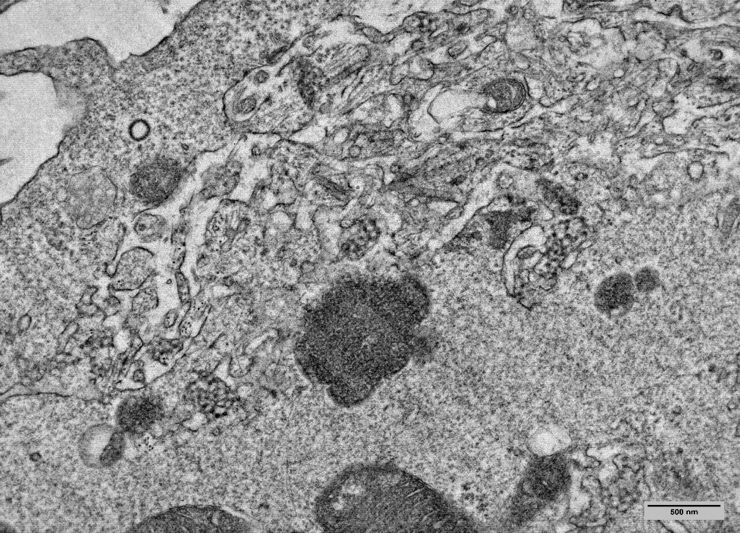


Vi

NS

NS


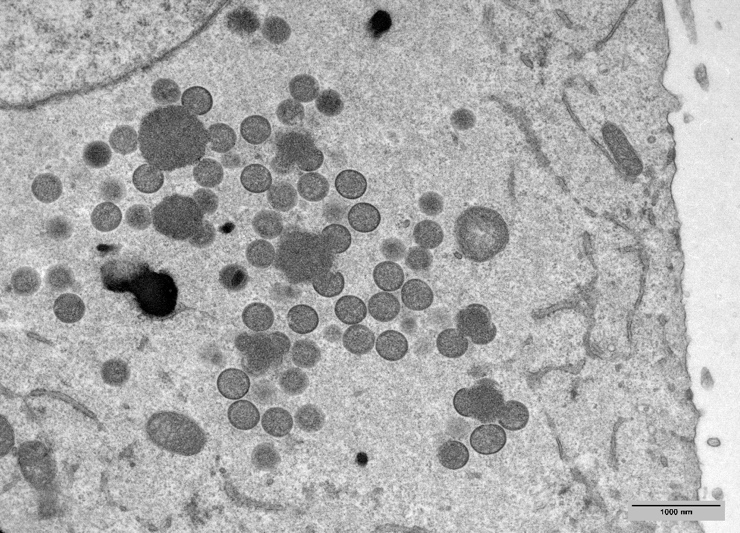


Vi

Vi

IV


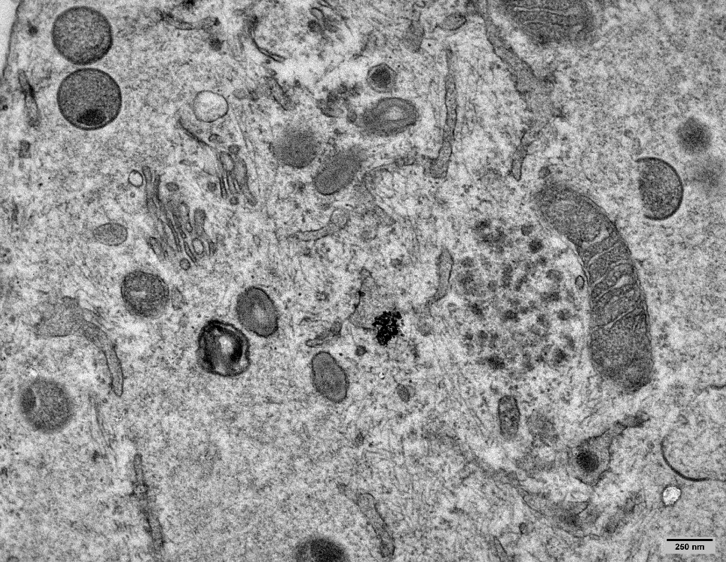


IV

MV


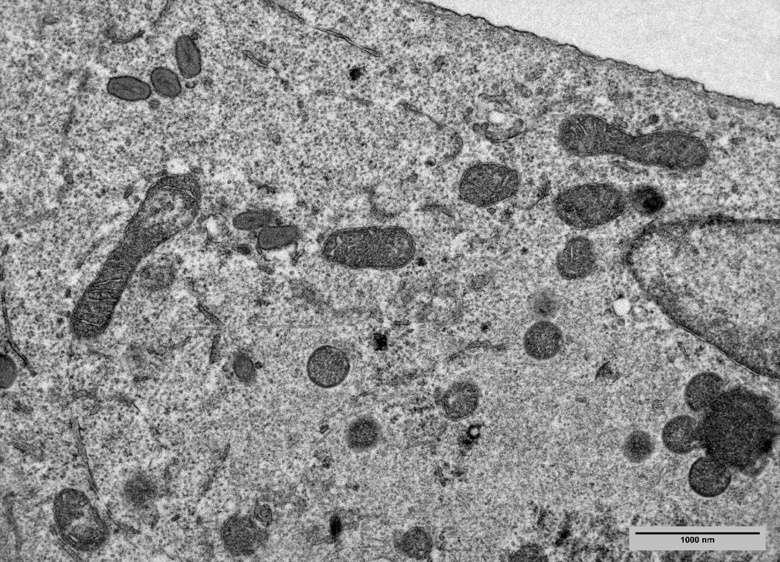


IV

MV

A

C

D

B

triple

full length

double

single


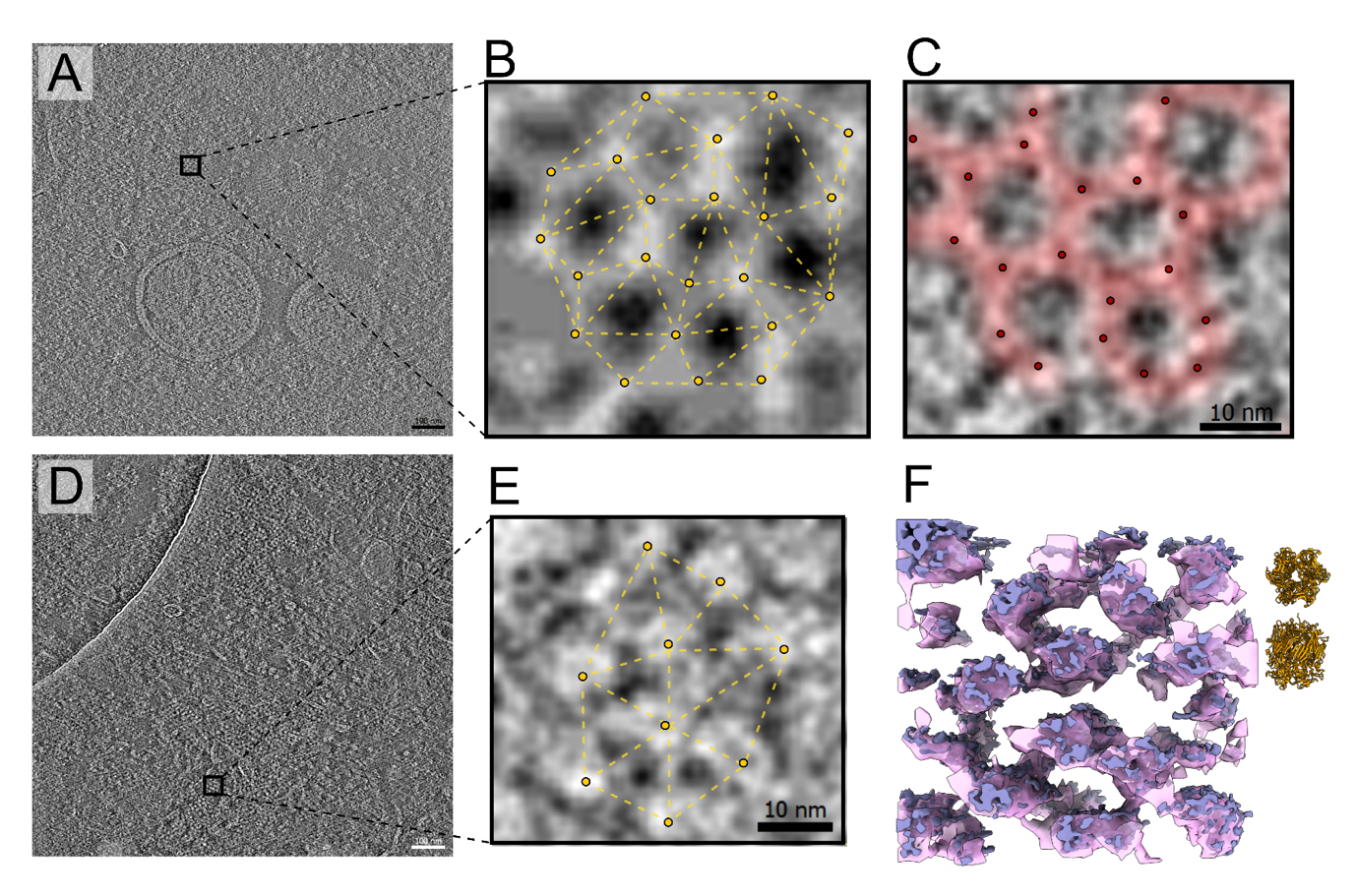

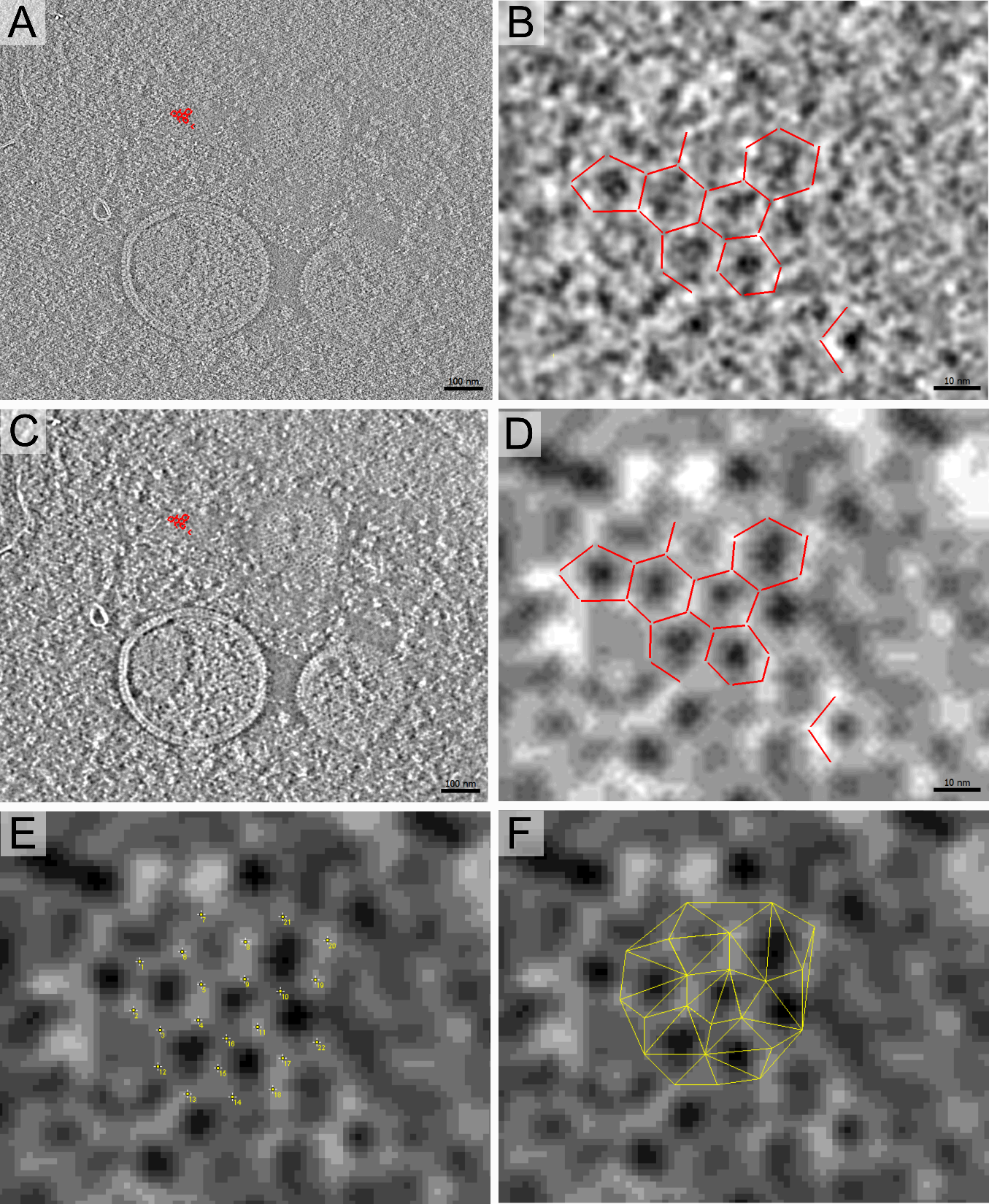


**Supplemental Figure 3. Honeycomb lattice in refrozen Tokuyasu sections**

HeLa cells were infected and prepared as for figure 2 and 70 nm thick sections immuno-labeled with anti-D13 followed by protein A coupled to 10 nm gold, prior to plunge freezing. A shows a slice of a tomogram of HeLa cells infected in the presence of IPTG (movie 5) with two IVs and a zoom (B and C) of the boxed area in A. The contrast is inverted for all images. In B and C the honey-comb pattern of D13 is apparent. In B the information is shown after application of the scale-space filter (Martinez-Sanchez *et al.,* 2014) to fully reveal the hexagonal arrangement of the D13 trimers (yellow dots in B, black dots in C, Supplemental Figure 3). D) a slice of a tomogram of HeLa cells infected without IPTG. A zoom of the boxed area in D is shown in E and F. Individual D13 trimers (yellow dots in E) located at a greater distance from each other (Delaunay triangulation distance = 13.75 ± 2.67 nm), failing to arrange in the typical honeycomb lattice (Delaunay triangulation distance = 10.80 ± 3.23 nm, Supplemental Figure 3). In F the D13 trimers are rendered in violet and pink based on their original intensities and their gaussian filter respectively using different isosurface thresholds. In the extreme upper right corner of F, two different orientations (top & side view) of the D13 atomic structure (pdb id: 2ygb) in yellow cartoon representation, as size reference.

**Supplemental figure 4: Assigning the D13 positions to the wild type honeycomb lattice.** A) A slice of the tomogram in Movie 5 (related to figure 5A to 5C). Red color marks the position of the IMOD model created. B) A zoom in that area. Red contours/lines represent the honeycomb lattice as seen from top. The positions of the D13 trimers were assigned based on the filtered tomogram as seen in panel D. C) The same slice as in A after application of the scale-space filter (Martinez-Sanchez *et al.,* 2014). D) The same zoomed area as in panel B after the scale-space filter. The IMOD modeling took place on this filtered volume and then overlayed back to the original volume in B. E) The same filtered zoomed volume as in D opened with ImageJ (Fiji) to assign the D13 trimers and F) calculate the Delaunay triangulation (Schindelin *et al.,* 2012).
